# Serum miR-146a and miR-150 as Potential New Biomarkers for Hip Fracture-Induced Acute Lung Injury

**DOI:** 10.1155/2018/8101359

**Published:** 2018-10-28

**Authors:** Li Gan, Tiansheng Sun, Bei Li, Jing Tian, Jianzheng Zhang, Xiaobin Chen, Jianfeng Zhong, Xiao Yang, Qi Li

**Affiliations:** ^1^Department of Orthopaedics, Zhujiang Hospital, Southern Medical University, Guangzhou, China; ^2^Department of Orthopaedics, PLA Army General Hospital, Beijing, China; ^3^Department of Orthopedics, Gaoming District People's Hospital of Foshan City, Foshan, China; ^4^Department of Spine Surgery, Second Affiliated Hospital of Xi'an Medical University, Xi'an, China; ^5^Department of Plastic and Reconstructive Surgery, Zhujiang Hospital, Southern Medical University, Guangzhou, China

## Abstract

**Background:**

Acute lung injury (ALI) and subsequent pulmonary infection are the most severe and usually fatal complications for elderly hip fracture patients. It is necessary to find some biomarkers for early diagnosis and prognosis of it.

**Objective:**

This study is aimed at examining the differential expression of miR-146a, miR-150, and cytokines (IL-6 and IL-10) between younger and elderly rats suffering from hip fracture and investigating the possible meaning of them in early diagnosis and prognosis of ALI after hip fracture.

**Methods and Subjects:**

Elderly rats and younger rats were randomly divided into sham group and fracture group, respectively. Two fracture groups received hip fracture operations. The damage degree of ALI was evaluated by histological observation and pathological score. Cytokines were measured by ELISA; miR-146a and miR-150 were analysed by qRT-PCR.

**Results:**

After treatment, compared with the corresponding sham groups, the pulmonary histological score, the serum miR-146a concentrations, and the cytokine (IL-6 and IL-10) levels in serum and BALF were significantly higher (the miR-150 were lower) in the fracture groups (with the exception of IL-6 of the younger fracture group at 72 h, all *P* < 0.05). Meanwhile, compared with the younger fracture group, the aforementioned variables were significantly higher (the miR-150 levels were lower) in the elderly fracture group (with the exception of serum IL-10 and pulmonary histological score at 8 h, all *P* < 0.05). The results of linear regression analysis showed that serum miR-146a and miR-150 were significantly associated with pulmonary histological score.

**Conclusion:**

Hip fracture can result in significant systemic inflammation and ALI in the rats. Compared to the younger rats, the elderly rats suffered a more remarkable ALI after hip fracture. It may be related to the abnormal expression of miR-146a and miR-150. Serum miR-146a and miR-150 are potential biomarkers for diagnosis and prognosis of ALI after hip fracture.

## 1. Introduction

Elderly hip fracture is a common clinical disease; it has a high mortality rate due to the significant excess complications after fracture [1, 2]. Among these complications, lung infection is the most serious and fatal [3]. Recently, more and more researches have indicated that pulmonary infection after elderly hip fracture is related to ALI, which resulted from the systemic inflammatory responses induced by the trauma of hip fracture [4–6]. The older the patients are, the higher the risk of pulmonary complications is; it leads to higher mortality [2, 3]. It was still uncertain why elderly patients suffer more easily from it and younger ones do not under almost similar clinical conditions. In order to reduce the incidence of pulmonary infection and decrease the mortality after hip fracture, it is very necessary to understand its underlying pathophysiological mechanisms and to timely detect and effectively treat the posttraumatic inflammation and lung injury. Traditional biomarkers of ALI induced by systemic inflammation mainly are inflammatory mediators (such as IL-6 and IL-10) in the serum and BALF [7–10]. However, inflammatory mediators are not specific enough due to an overlap with other inflammatory diseases [11–13]. Therefore, ideal biomarkers with higher sensitivity and specificity are remained to be identified.

MicroRNAs (miRNAs) are small noncoding RNAs that regulate gene expression and play important roles in a variety of cellular functions [14, 15]. Some miRNAs have been identified in serum and plasma as biomarkers for several diseases [16]. Recently, miR-146a and miR-150 are identified as potential biomarkers of sepsis and play a role in regulating inflammation [17–19]. However, their roles in systemic inflammatory responses and ALI after trauma remain uncertain. The purpose of our study was to investigate the serum levels of miR-146a and miR-150 in rats that suffered hip fracture and to identify the relationship between them and ALI induced by hip fracture.

## 2. Materials and Methods

### 2.1. Grouping of Animals

Rats of 22–23 months old are considered elderly [20]. 40 elderly male Sprague Dawley (SD) rats (age: 22–23 months, Animal Experiment Center of Southern Medical, University, China) and 40 younger male SD rats (age: 8–9 months, Animal Experiment Center of Southern Medical University, China) were randomly divided into two groups (sham group and fracture group), respectively, after being allowed to acclimate for 1 week. The elderly sham group (*n* = 20) and the younger sham group (*n* = 20) only received anesthesia, cannulation, and observation. The elderly fracture group (*n* = 20) and the younger fracture group (*n* = 20) also received hip fracture operations in addition to the above. Experiments were performed according to the guidelines for experimental animal care and use approved by the Southern Medical University.

### 2.2. Fracture Model

A total of 40 rats (20 elderly and 20 younger) were anaesthetised, respectively, with 10% chloral hydrate (3.5 ml/kg, i.p.) and then placed in a prone position on the base of a blunt guillotine ramming apparatus. The rats were fixed after the proximal femur was identified and marked. The 500 g blunt guillotine was lifted to a 15 cm height and was allowed to fall freely along the axis. The force of the falling object resulted in a unilateral closed proximal femoral fracture (hip fracture). X-ray confirmed the fracture.

### 2.3. Collection of Samples

The rats, respectively, were sacrificed at 8, 24, 48, and 72 h after treatment. The thoraxes were opened rapidly; the blood samples were collected by heart puncture for later miRNAs and cytokine assay. Lung tissues were quickly harvested, the left trachea was exposed, and the bronchoalveolar lavage fluid (BALF) was collected three times through a tracheal cannula with autoclaved PBS that was instilled up to a total volume of 1.0 ml and then centrifuged at 3000 rpm for 10 min. The supernatant was frozen at −80°C until the subsequent cytokine assays. The right lung was fixed immediately in 10% formalin and stored at 4°C for subsequent histological observation and pathological scoring.

### 2.4. Histological Analysis

The lung specimens were fixed with 10% formalin and embedded in paraffin. Tissue sections (5–8 *μ*m) were prepared and stained with haematoxylin and eosin (H&E). Briefly, 3 slices were randomly selected from each rat, and the three fields of each slice were reviewed under a microscope (100× magnification, Olympus DP71, Tokyo, Japan). All slides were examined and scored by an experienced pathologist (Xiao Yang) who was blinded to the experimental groups based on the lung injury scoring system [21]. The score was based on categories of inflammatory cell infiltration, pulmonary oedema, congestion, and intra-alveolar hemorrhage that were graded on a scale of normal (0), mild (1), moderate (2), or severe (3) injury, with a maximum possible score of 12.

### 2.5. Cytokine Assay

After the blood samples were collected, the serum was separated immediately and stored at −80°C. Plasma concentrations of IL-6 and IL-10 were measured by the ABC enzyme-linked immunosorbent assay (ELISA) system (R&D Systems, USA) following the manufacturer's instructions.

### 2.6. Serum miRNA Isolation

The serum was extracted before the blood samples were incubated at 37°C for 1 h and centrifuged at 2500 rpm for 10 min. Serum miRNA was isolated by TRIzol reagent (Promega, USA) and a miRcute miRNA extraction kit (Tiangen, China) according to the manufacturers' protocol.

### 2.7. qRT-PCR

A 1–4 ng aliquot of total RNA was then subjected to cDNA synthesis using either miRNA- (miR-150- and miR-146a-) or U6 snRNA-specific primers. 5S rRNA amplifications using hexamer-primed cDNA as template were performed as positive controls. All reactions were performed using the miRCURY LNA™ microRNA PCR system (Exiqon, Denmark) according to the manufacturer's instructions [22]. The cycling conditions were 95°C for 10 min, followed by 42 cycles at 95°C for 20 s and 60°C for 1 min. All cycles were completed on an Mx3005P cycler (Stratagene, USA). The miRNA expression levels were measured using SYBR green qRT-PCR, normalised to those of U6 snRNA, and calculated by the comparative CT method.

### 2.8. Statistical Analysis

Data were presented as means ± standard deviation. All data were analysed by SPSS software (version 13.0). One-way ANOVA with Bonferroni post hoc tests were performed to compare the data of all groups at each monitoring point. Quantitative data were compared between two groups using *t*-test. In order to evaluate the predictive value of miRNAs (miR-146a and miR-150) and cytokines (IL-6 and IL-10), linear regression analysis was applied to measure the relationships between pulmonary histological score and them. A *P* value of less than 0.05 was considered to be statistically significant.

## 3. Results

### 3.1. Hip Fracture-Induced Changes of IL-6, IL-10, miR-146a, and miR-150 Levels in the Serum

**Figure 1:**
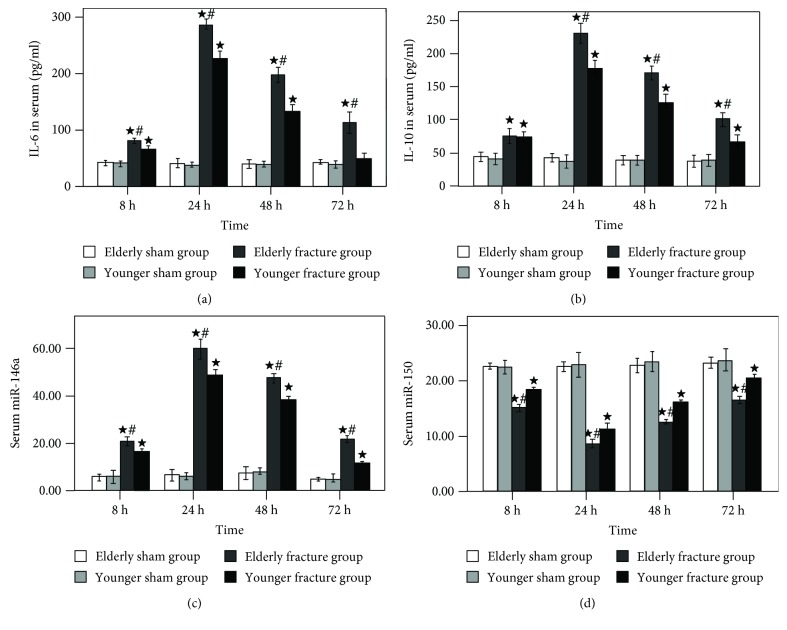
Time course of serum IL-6 (a), IL-10 (b), miRNA-146a (c), and miRNA-150 (d) levels. Results are expressed as mean ± standard error. Filled star: *P* < 0.05 for the elderly fracture group versus the elderly sham group and the younger fracture group versus the younger sham group. Pound sign: *P* < 0.05 for the elderly fracture group versus the younger fracture group.

As shown in Figure 1, serum concentrations of IL-6 (Figure 1(a)), IL-10 (Figure 1(b)), and miR-146a (Figure 1(c)) began to increase gradually after hip fracture, peaked at 24 h, and then gradually declined. Contrarily, serum miR-150 levels (Figure 1(d)) of the fracture groups began to descend gradually after hip fracture, reached the minimum at 24 h, and then gradually rose.

At 8, 24, 48, and 72 h after treatment, the serum concentrations of cytokines (IL-6 and IL-10), miR-146a, and miR-150 had no significant difference between the elderly sham group and the younger sham group; however, compared with the corresponding fracture groups (elderly sham group vs. elderly fracture group, younger sham group vs. younger fracture group), the serum cytokine (IL-6 and IL-10) and miR-146a levels were significantly higher (the miR-150 levels were lower) in the fracture groups (with the exception of serum IL-6 levels of the younger fracture group at 72 h, all *P* < 0.05). Meanwhile, compared with the younger fracture group, the aforementioned variables were significantly higher (with the exception of the miR-150 levels which were lower) in the elderly fracture group (with the exception of serum IL-10 levels at 8 h, all *P* < 0.05).

### 3.2. Hip Fracture-Induced Pulmonary Changes

**Figure 2:**
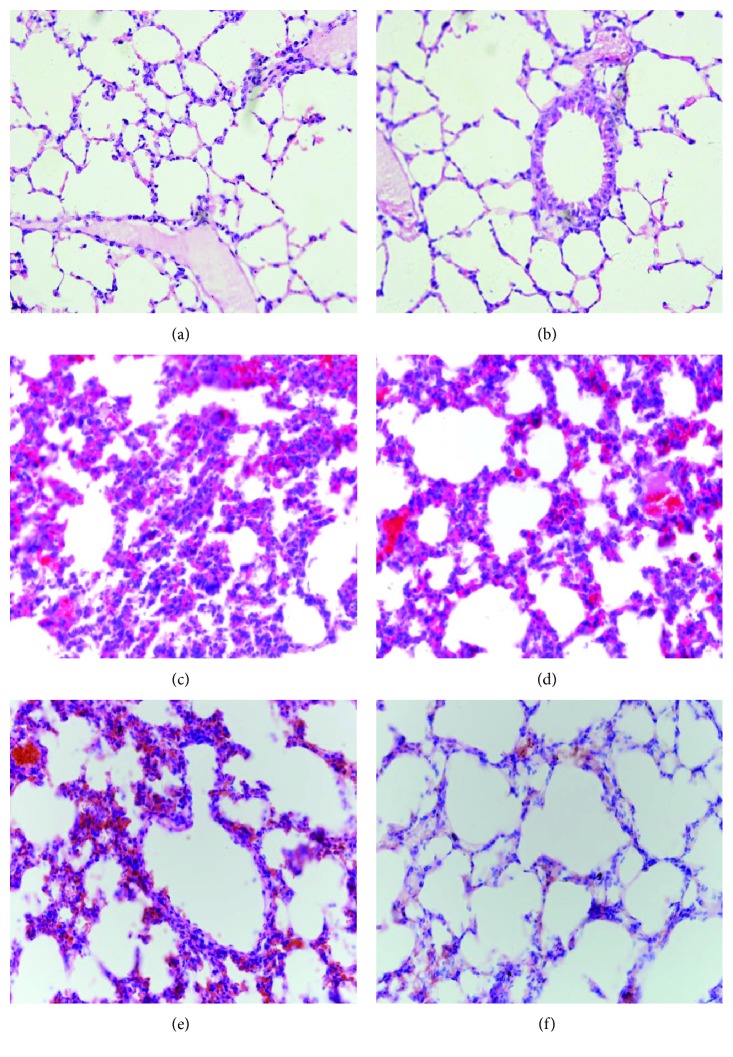
Representative H&E sections of pulmonary tissues (magnification: 200×). At 24 h after treatment, the elderly sham group (a) and the younger sham group (b) had no obvious inflammation; however, the elderly fracture group (c) and the younger fracture group (d) show typical manifestations of acute lung injury. At 72 h after hip fracture, the lung inflammatory manifestations of younger fracture group (f) had subsided, but it was still obvious in the elderly fracture group (e).

As shown in Figures 2(a) and 2(b), the tissue slices of the elderly sham group and the younger sham group had no histopathological signs of ALI after treatment. However, as shown in Figures 2(c) and 2(d), the tissue slices of the elderly fracture group and the younger fracture group had obvious histopathological signs of ALI (pulmonary edema, congestion, polymorphonuclear and mononuclear cell infiltrates, and damaged alveolar architecture) after hip fracture. At 72 h after treatment, the lung inflammatory manifestations of the younger fracture group (Figure 2(f)) significantly subsided and were similar to the sham group (Figure 2(b)) with the exception of some neutrophil infiltrates, but the pulmonary inflammation of elderly fracture group (Figure 2(e)) was still obvious.

**Figure 3:**
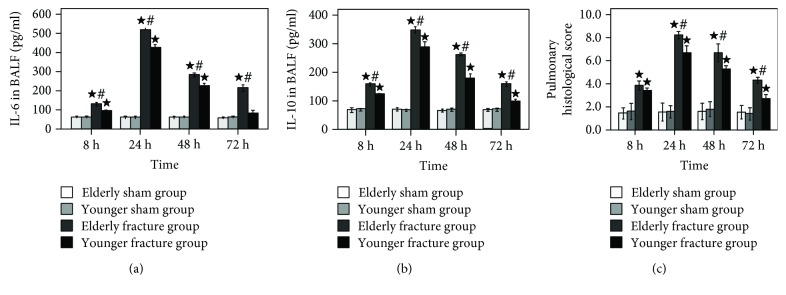
Time course of pulmonary histological score (c) as well as IL-6 (a) and IL-10 (b) concentrations in BALF. Results are expressed as mean ± standard error. Filled star: *P* < 0.05 for the elderly fracture group versus the elderly sham group and the younger fracture group versus the younger sham group. Pound sign: *P* < 0.05 for the elderly fracture group versus the younger fracture group.

At 8, 24, 48, and 72 h after treatment, the IL-6 (Figure 3(a)) and IL-10 (Figure 3(b)) levels in the BALF as well as the pulmonary histological score (Figure 3(c) and Table 1) had no significant difference between the elderly sham group and the younger sham group; however, compared with the corresponding fracture groups (elderly sham group vs. elderly fracture group, younger sham group vs. younger fracture group), these variables were significantly higher in the fracture groups (with the exception of BALF IL-6 levels of the younger fracture group at 72 h, all *P* < 0.05). Meanwhile, compared with the younger fracture group, the aforementioned variables were significantly higher in the elderly fracture group (with the exception of pulmonary histological score at 8 h, all *P* < 0.05).

### 3.3. The Results of the Linear Regression Analysis

**Figure 4:**
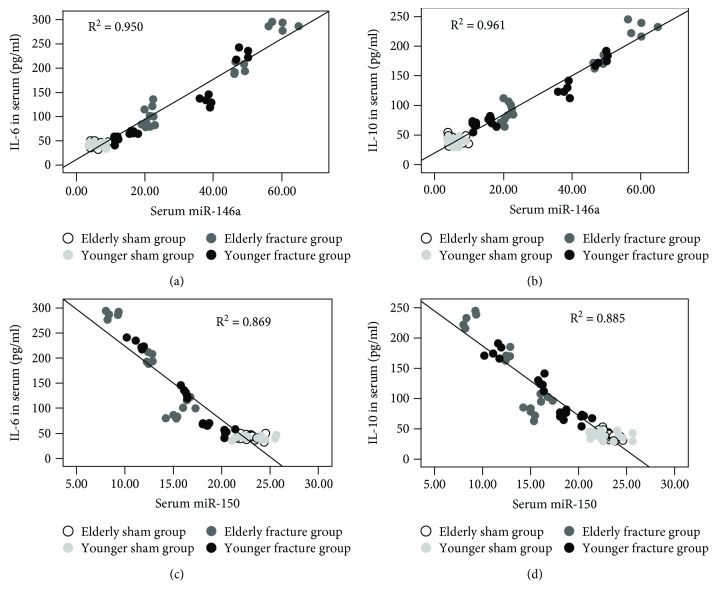
Scatter plots and regression lines showed that the serum miRNA-146a (a, b) and miRNA-150 (c, d) levels exhibited a significant relationship with the serum IL-6 and IL-10 concentrations.

**Figure 5:**
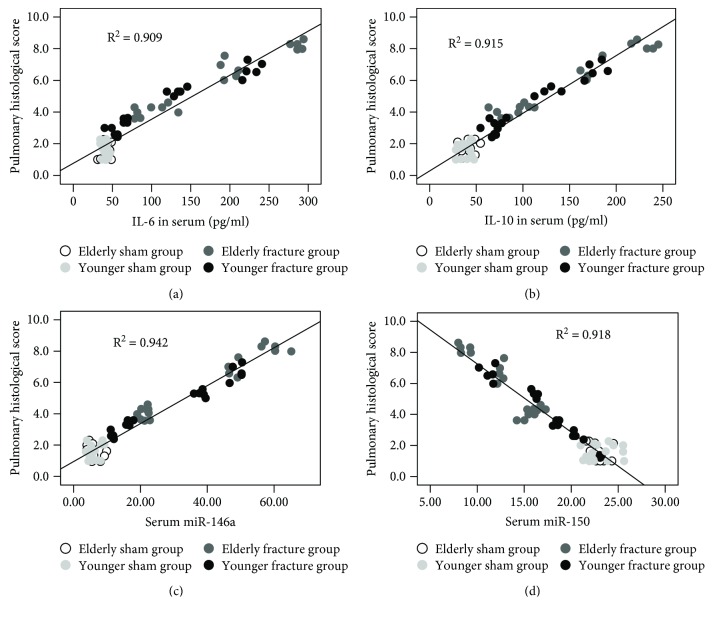
Scatter plots and regression lines showed that the serum IL-6 (a), IL-10 (b), miRNA-146a (c), and miRNA-150 (d) levels exhibited a significant relationship with the pulmonary histological score.

The results of the linear regression analysis revealed that the serum miR-146a and miR-150 levels exhibited a significant relationship with the serum IL-6 (*P* = 0.03, *B* = 0.081 and *P* = 0.045, *B* = −0.015) and IL-10 (*P* ≤ 0.001, *B* = 0.193 and *P* = 0.001, *B* = −0.058) concentrations (Figure 4) and the serum IL-10 (*P* = 0.003, *B* = 0.018), miR-146a (*P* = 0.004, *B* = 0.050), and miR-150 (*P* = 0.001, *B* = −0.125) levels were significantly correlated with the pulmonary histological score (serum IL-6 were removed, *P* = 0.240). Compared with serum IL-6 and IL-10, the serum miR-146a and miR-150 had more close relationship with pulmonary histological score (Figure 5).

## 4. Discussion

It is indisputable that trauma can trigger an inflammatory response [23]. Under normal conditions, organisms can restrict the inflammation induced by trauma locally. The local inflammation is beneficial to necrotic material removal and damage tissue repair. However, when the organisms are overwhelmed by the trauma, the local inflammation is out of control and result in systemic inflammatory response which leads to ALI [24–27]. The lung barrier function damage increases the susceptibility of organisms to pathogenic microorganisms; it leads to an increased incidence of pulmonary infection complication [28–30]. As the elderlies possess a reduced physiological reserve and are more vulnerable to posttraumatic inflammatory reaction, even a minor trauma can cause uncontrolled systemic inflammatory response and ALI [31–33]. It is consistent with our experimental results.

In our experiment, the pulmonary histological score and the cytokine (IL-6 and IL-10) levels in serum and BALF were significantly increased in the rats that suffered hip fracture. It prompts that hip fracture can result in significant systemic inflammation and ALI in the rats. Compared with the younger fracture group, the pulmonary histological score and the cytokine (IL-6 and IL-10) levels in serum and BALF were higher in the elderly fracture group. It means that the elderly rats have suffered more serious lung injury and systemic inflammation than the younger ones under the same degree of trauma. At 72 h after fracture, as shown in the histological sections, the lung inflammatory manifestations of the younger fracture group had significantly subsided; meanwhile, the cytokine (IL-6) levels in serum and BALF had no significant difference between the younger fracture group and the younger sham group. However, the pulmonary inflammation (according to the pulmonary histological score and the cytokine (IL-6 and IL-10) levels in BALF) and the systemic inflammation (according to the cytokine (IL-6 and IL-10) levels in serum) were still obvious in the elderly fracture group. It shows that the younger rats have almost recovered from systemic inflammation and lung injury at 72 h after hip fracture, while these pathological manifestations of the elderly rats are still obvious. The above results seem to imply that the elderly rats have suffered more serious posttraumatic inflammation and are more difficult to recover from the pathological state than the younger ones under the same degree of trauma. Maybe this is the reason why elderly patients with hip fracture more easily suffer a high incidence of complications and mortality. Its particular mechanism still needs further study.

More and more researches have shown that elderly hip fracture induced systemic inflammatory response and ALI, which increased the incidence of pulmonary infection complications and mortality in the elderly patients [2, 5, 6, 33, 34]. Although many measures have been taken to deal with it, such as perioperative respiratory assessment and management, surgical technique amendment, and internal fixation instrument improvement, pulmonary infection rates still remain high in clinic; it is still the main cause of death in the elderly fracture patients [35–37]. As the important cause of lung infection complications, systemic inflammatory response and ALI induced by the elderly hip fracture have attracted more and more attention. Early diagnosis and evaluation of posttraumatic lung injury are crucial for timely correction of this complicated syndrome. The potential biomarkers for posttraumatic lung injury include acute phase protein, cytokines, and chemokines [7–10]; however, all of which are not specific enough due to an overlap with other inflammatory diseases [11–13]. Therefore, ideal biomarkers with higher sensitivity and specificity remained to be identified.

Recent researches implied that serum miRNAs might be used as biomarkers for systemic inflammatory reaction [38, 39]. The studies of miR-146a indicated that miR-146a was in control of inflammation during the innate immune response through a negative feedback regulation loop involving downregulation of Toll-like receptor and cytokine signaling [40, 41]. The studies on miR-150 shown that it played important roles in the innate immune response through the regulation of NK, B, and T cell development, maturation, and function [42–44]. Some research results demonstrated that miR-146a and miR-150 expressions differentiated sepsis patients from healthy controls and might serve as biomarkers of sepsis [45, 46]. In our study, the hip fracture not only induced abnormal expression of cytokines (IL-6 and IL-10), miR-146a, and miR-150 but also caused ALI; the traditional inflammatory biomarker (IL-6) was not significantly related with evaluation indicator (pulmonary histological score) of ALI. However, serum miR-146a and miR-150 levels were significantly associated with pulmonary histological score and had more close relationship with it (compared with serum IL-10). It implies that serum miR-146a and miR-150 may be the potential biomarkers of ALI after hip fracture.

In general, hip fracture induced remarkable cytokine (IL-6 and IL-10) release, miRNA (miR-146a and miR-150) abnormal expression, and ALI. After hip fracture, the elderly rats were more prone to these pathological states than the younger ones. Compared with cytokines (IL-6 and IL-10), serum miR-146a and miR-150 had more close relationship with ALI following hip fracture. Serum miR-146a and miR-150 may serve as biomarkers for the diagnosis and prognosis of ALI induced by hip fracture.

In this study, there are some limitations that need to be declared. (1) We only got preliminary experiment results that elderly rats suffer more serious systemic inflammation and ALI than the younger ones under the same degree of trauma. For this particular mechanism of elderly rats vulnerable to systemic inflammation and ALI after fracture, we did not have an in-depth study. (2) We identified that serum miR-146a and miR-150 were significantly associated with pulmonary histological score and speculated they may be the potential new biomarkers for hip fracture-induced acute lung injury; however, we did not delve into the particular mechanisms of miR-146a and miR-150 in the posttraumatic inflammation. (3) Because of the difference between experimental animal models and clinical patients, the results of this research may not be identical to the clinical situation. It remains to be determined whether serum miR-146a and miR-150 should be introduced into clinical practice for prediction of ALI following trauma. (4) There may be a few flaws in the design of this experiment; improving inspection method of miRNAs may make the experiment more perfect. In order to answer these questions, further study is necessary.

## Figures and Tables

**Table 1:** The results of pulmonary histological score.

Group	Pulmonary histological score $\left(\bar{x}\pm s\right)$			
	8 h	24 h	48 h	72 h
Elderly sham group	1.44 ± 0.38	1.78 ± 0.87	1.64 ± 0.76	1.64 ± 0.65
Younger sham group	1.78 ± 0.87	1.90 ± 0.62	1.58 ± 0.44	1.58 ± 0.83
Elderly fracture group	3.30 ± 0.54^a^	6.58 ± 1.07^ab^	4.90 ± 0.59^ab^	4.50 ± 0.50^ab^
Younger fracture group	2.78 ± 0.40^a^	5.92 ± 0.30^a^	4.30 ± 0.21^a^	3.10 ± 0.31^a^

^a^ *P* < 0.05 for the elderly fracture group versus the elderly sham group and the younger fracture group versus the younger sham group at the same time point. ^b^*P* < 0.05 for the *x*elderly fracture group versus the younger fracture group at the same time point.

## Data Availability

The data used to support the findings of this study are available from the corresponding author upon request.
